# Resilience of research capacity strengthening initiatives in Africa during crises: the case of CARTA during COVID

**DOI:** 10.1080/16549716.2023.2240153

**Published:** 2023-08-10

**Authors:** Florah Karimi, Marta Vicente-Crespo, Mercy Ndwiga, Naomi Njenga, Rita Karoki, Sharon Fonn

**Affiliations:** a Division of Research and Related Capacity Strengthening, African Population and Health Research Center, Nairobi, Kenya; b School of Public Health, University of the Witwatersrand, Johannesburg, South Africa; c School of Public Health and Community Medicine, University of Gothenburg, Gothenburg, Sweden

**Keywords:** Research capacity strengthening (RCS) initiatives, resilience during crises, interventions, enablers of success, consortium for advanced research training in Africa (CARTA)

## Abstract

**Background:** Several research capacity strengthening (RCS) initiatives have been established in Africa over the past decade. One such initiative is the Consortium for Advanced Research Training in Africa (CARTA) that has gained traction over the years and has been proven as an effective multidisciplinary approach to strengthen research capacity to address public and population health in Africa. **Objectives:** In this article, we document the experiences and management-related interventions that cushioned the CARTA programme and enabled it to remain resilient during the COVID pandemic. We further make recommendations on the enablers of resilience and optimal performance of such RCS initiatives during crises and beyond. **Methods**: We used routine information gathered by the CARTA secretariat from consortium correspondence, meeting minutes, reports and other related documents produced in the year 2020 in order to consolidate the experiences and interventions taken by the programme at programmatic, institutional and fellowship levels. **Results:** We identified a series of management-related cyclic phases that CARTA went through during the pandemic period, which included immobilisation, reflection, brainstorming, decision-making, intervening and recovery. We further identified strategic management-related interventions that contributed to the resilience of the programme during the pandemic including assessment and monitoring, communication management, policy and resource management, making investments and execution. Moreover, we observed that the strength of the leadership and management of CARTA, coupled with the consortium´s culture of collaboration, mutual trust, respect, openness, transparency, equitability, ownership, commitment and accountability, all contributed to its success during the pandemic period. **Conclusion:** We conclude that RCS initiatives undergo a series of phases during crises and that they need to promptly adopt and adapt appropriate management-related strategic interventions in order to remain resilient during such periods. This can be significantly realised if RCS initiatives build a culture of trust, commitment and joint ownership, and if they invest in strong management capacity.

## Introduction

In 2020, the economic and social progress of the African region was threatened by COVID pandemic-related disruptions [1]. It became crucial to prioritise transformational and innovative thinking on preparedness, responsiveness and resilience [2] and to mitigate against adverse effects of the pandemic on livelihoods and development. During this period, African countries advocated for, and employed, a number of mitigation measures, including testing, case management and control measures, in line with advisories received from the World Health Organization (WHO) [3]. Despite the mitigation measures adopted, economic and social development within Africa was still hampered by low testing capacity and structurally weak health systems [1,4]. Regrettably, preparedness to respond to crises, including pandemics, has remained wanting in Africa. Part of the inability to mount an adequate response was attributed to weak research systems, including a lack of conducive environments for research and innovation [3,5].

Over the past 10 years, several research capacity strengthening (RCS) initiatives have been established in Africa [6], which aim to address the inadequacies of conducive research environments within the region. These initiatives range from the provision of individual training and fellowship support to institutional and systemic support [7]. The Consortium for Advanced Research Training in Africa (CARTA) is one of the African RCS initiatives that has been proven to be effective [8–10] and remained resilient during the COVID pandemic [3].

### Description of the CARTA programme

This section provides an overview of how CARTA was set up and how it operates. CARTA was established in 2008 as an Africa-based, Africa-led initiative to rebuild and strengthen the capacity of African universities to produce well-trained and skilled researchers and scholars in Africa [9]. It evolved as a result of African researchers who had similar RCS interests, seeking to set up a coordinated joint PhD training and institutional-strengthening programme [11]. This was in recognition of the fact that, while universities were well positioned to contribute to development agendas through the provision of skilled human resources and evidence that informs decision-making processes at national, regional and global levels, they lacked the capacity to do so in the African region.

CARTA was therefore established through a South–South partnership of African universities and research institutions, with South-North collaboration of non-African universities and research institutions to address the research capacity need within the region [9]. Box 1 provides additional information on the conceptualisation of CARTA.

**Table:** **Box 1:** Conceptualisation of CARTA.

The conceptualisation of CARTA was catalysed during a meeting convened by the African Population and Health Research Center (APHRC), in response to a ‘Wellcome Trust African Institutions Initiative’ Call. Institutions that were interested and able were invited to determine the overall purpose of CARTA, its management structure, including the lead institutions, and its accountability mechanisms. The African institutions were further invited to be part of the consortium. Those that responded and met the basic requirements for the Call were incorporated into the consortium. The consortium subsequently invited and incorporated non-African partners with whom they had previous mutual beneficial research relationships to join in. The consortium established a full-time secretariat, based at APHRC, which was also be the legal entity for CARTA.

It is worth noting that CARTA’s African research institutions (both independent and satellites of bigger universities) were incorporated from its inception, in recognition of the need to also build their research capacity and have them contribute to the shared experience within the programme. The non-African institutions supplement African capacity by sharing experiences of working in research-intensive environments often coupled with doctoral degree programmes and providing additional mentorship capability.

**Figure 1.**
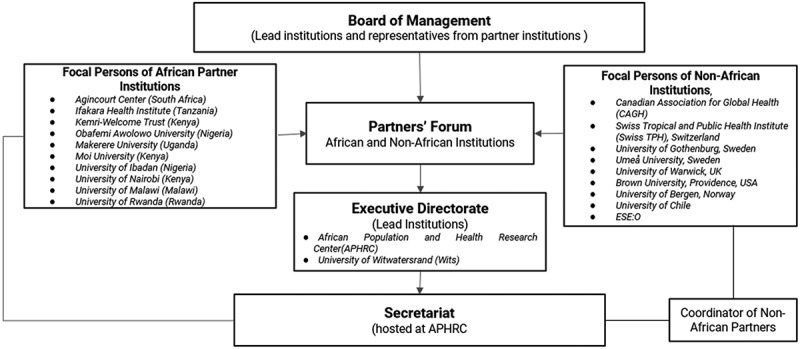
CARTA partner institutions and governance structure.

CARTA is hosted at the African Population and Health Research Center (APHRC) in Kenya that also co-directs the programme with the University of the Witwatersrand (Wits) in South Africa, in partnership with the African and non-African partner universities and research institutions. The relationship between CARTA’s partner institutions and related governance structure is presented in Figure 1. The partnership and governance operations of CARTA are further describe in Box 2.

**Table:** **Box 2:** How CARTA works.

Each member institution has at least one designated CARTA focal person. These focal people are nominated by the Vice Chancellor or institutional head of research institutions. Through the focal people, institutions are represented at the annual Partners Forums where the performance and progress of the programme are monitored and new strategies, initiatives, policies and annual plans are discussed. Proposals from the Partners Forum are passed on to the Board of Management (BoM), which takes responsibility for strategic and policy-related decisions of the programme. The Consortium also holds regular meetings with the Vice Chancellors or institutional heads of research institutions. The day-to-day work of CARTA is carried out by a secretariat, located at APHRC, and is managed by the Executive Directorate, which comprises the heads of the two lead institutions and the head of research capacity building at APHRC.

The goal of CARTA is to improve African public health and population wellbeing through stimulating excellent multidisciplinary research in African public universities and research institutions, and evidence uptake (https://cartafrica.org/). Upon structuring the consortium and determining the partnership rules and the mission and vision of the programme, CARTA, during its first phase (2011 to 2015), focused on strengthening doctoral training through the creation of a collaborative doctoral training programme in public and population health, and the research infrastructure and capacity of African partner institutions to provide a more research-conducive environment. CARTA expanded its focus during its second phase (2016 to 2020) to include two more strategic thrusts – one to secure the future of CARTA graduates, and the second to share its learning with the African partner institutions through a CARTA institutionalisation strategy.

**Figure 2.**
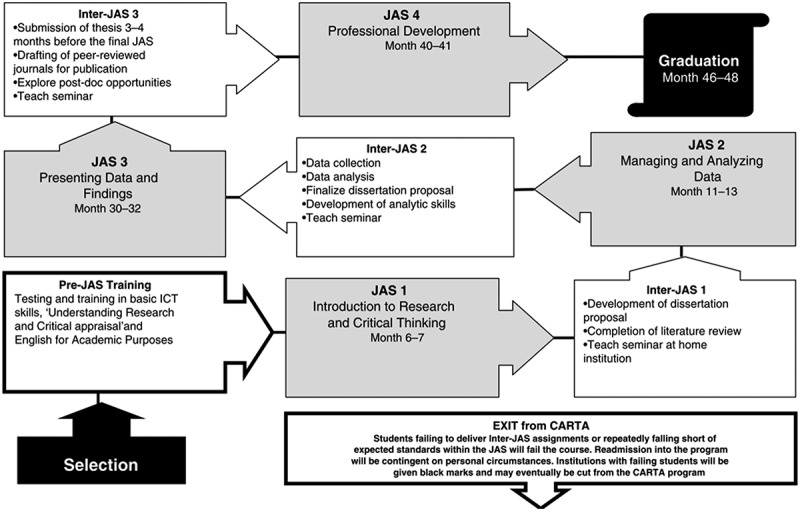
Fellows’ progression milestone [12].

According to the CARTA Theory of Change that is described in Box 3, CARTA prioritises the development of high-quality research leaders for the African region through its enriched fellowships that are supported by research supportive and responsive institutions. It convenes four hierarchical compulsory Joint Advanced Seminars (JAS) for all its fellows, which are held as residential sessions hosted at selected African partner universities and aligned to the fellows’ progression milestones illustrated in Figure 2.

**Table:** **Box 3:** CARTA’s Theory of Change.

CARTA’s Theory of Change (ToC) is hinged on inter-generational transfer of skills and culture, and a critical mass of change makers within each university. It prioritises skilled early career researchers, established research teams, conducive training and research environments, and quality scientific and engagements as outcomes that influence research leadership and strengthened sustained training and research.

Between 2011 and 2020, CARTA had supported 245 fellows in their PhD journey, with 97 of them graduating from African partner universities and taking up 72 postdoctoral opportunities within this period [9]. Furthermore, CARTA fellows and graduates had published 1652 articles in peer-reviewed journals, as demonstrated on the CARTA website https://carta-evidence.org/bibliometric-dashboard/), advancing the African public and population health research agenda [13] through collaborative multidisciplinary research [14].

### Purpose of the article

Very little has been documented about the levels of preparedness, responsiveness and resilience of RCS initiatives in Africa during crises. In this article, we consider how CARTA remained resilient during the COVID pandemic, highlighting the experiences of the programme and the management-related interventions that cushioned it and enabled it to remain on course during this period. This article also offers recommendations on the enablers of resilience and optimal performance of such RCS initiatives during crises and beyond.

## Methodology

This article is descriptive in nature and uses routine data extracted by the CARTA secretariat during the COVID pandemic period. The information was extracted by five secretariat members from e-mail correspondence, minutes of meetings, status and monitoring and evaluation reports produced between February and December 2020. This was compiled into an Excel spreadsheet. The extraction tool allowed us to collect information under various variables, as presented in Table 1.

**Table 1.** Variables extracted from the correspondence and documents produced by CARTA.

No.	Variables
1	Date of occurrence of the events.
2	Type of correspondence – extracts from documents, minutes of meetings, electronic correspondence, or reports.
3	Source from where the information was obtained – all stakeholders, secretariat, Executive Directorate, institutional focal persons, vice chancellors, Board of Management, coordinators and facilitators of interventions, fellows, funders, travel agency, digitisation consultant and APHRC leadership.
4	Target groups for the information - all stakeholders, secretariat, Executive Directorate, institutional focal persons, Board of Management, coordinators and facilitators of interventions, fellows, funders, travel agency, hotels and APHRC leadership.
5	Brief summary on the type of content area of the information.
6	Experiences encountered, including challenges, and responses elicited.

In a first round of extraction, each of the five members extracted information related to communication and meetings in which they were involved. Since the secretariat works in a collaborative manner and there is no communication or meeting in which only one member is involved, all members reviewed the spreadsheet to add details, detect gaps and corroborate the accuracy of the data extracted. Thus, we collectively validated the information and further synthesised it for ease of analysis.

We analysed the themes that arose and classified them as experiences or responses, by month and at varying levels – programmatic, institutional and individual/fellowship. The aim of the monthly segregated data was to establish possible patterns of responses from the time COVID was considered a global pandemic until the end of 2020.

## Results

### Experiences of the programme during the COVID pandemic

The CARTA programme was affected by the COVID pandemic in several ways, and at the three interlinked programmatic, institutional and individual/fellowship levels.

#### Programmatic-level experiences

CARTA’s scheduled activities for the year, including governance meetings and RCS workshops and seminars, were disrupted due to travel restrictions and enhanced health protocols that limited face-to-face engagements at the onset of the pandemic. Table 2 presents the scheduled programmatic activities for 2020 and the adjustments made to ensure that they were not adversely affected by the pandemic.

**Table 2.** Scheduled 2020 CARTA programmatic activities and adjustments made amidst the pandemic.

Implications of COVID on the CARTA 2020 scheduled activities			
Scheduled Activity	Scheduled Dates	Venue	COVID-related adjustments
JAS 1 for Cohort 10 JAS 4 Cohort 7	March 2 -26	Makerere University, Uganda	Shortened the face-to-face residential sessions to enable the participants travel back to their countries before boundary restrictions Held online sessions for the remaining sessions
Vice Chancellors’ Meeting	Not scheduled for 2020	Virtual meeting	Held an emergency virtual Vice Chancellors’ meeting in April 2020 to
			establish how each institution had been affected by the pandemic determine how each institution was responding to the situation at hand determine the progress being made to provide support to post-graduate research programs identify how CARTA-funded PhD students and post-doctoral fellows continue with their work.
21^ST^ CARTA Board of Management Meeting	March 26	Makerere University, Uganda	Held virtually
Faculty and Administrators Workshop	June 22 - 25	University of Malawi, Malawi/APHRC, Nairobi	Held as a mini virtual workshop that focused on strengthening only one research-support functionary area (grant management) in partner institutions rather than the entire spectrum of functionary areas including library, enrolment, financial and accounts, Information, Communication and Technology (ICT), and research management support.
JAS 3 Cohort 8	August 3 - 28	University of Ibadan, Nigeria	Rescheduled to be held in 2021 adopting a blended learning approach that comprised virtual, in-country residential, and clinic (one-on-one diagnostics) sessions, and spread over longer durations of time, as compared to the regular four-week face-to-face sessions
4^th^ Focal Persons Forum	September 21	APHRC, Nairobi	Rescheduled to August 2020 as a virtual meeting to:
			apprise on institutional experiences resulting from COVID brainstorm on how to maximize the utilization of resources and ensure accounting of funds before close up of projects, and the way forward on how to enhance the gains of the program in institutions.
11^th^ CARTA Partners’ Annual Forum	September 22-23	APHRC, Nairobi	Held virtually with a focus on the implications of COVID-19 on the CARTA program and the new normal.
22^nd^CARTA Board of Management and Funders’ Meetings	September 24	APHRC, Nairobi	Held virtually with a focus on the implications of COVID-19 on the CARTA program and the new normal.
CARTA 10 YEARS Scientific Conference	September 25-26	APHRC, Nairobi	Re-programmed as 10 years over a six-month period (from July to December 2020) in 2020. Included social media, articles on news outlets, webinars and a scientific conference.
JAS 2 Cohort 10	November 2-26	University of the Witwatersrand, South Africa	Offered in two parts The first part 2 was offered virtually for four weeks in November 2020 and included virtual sessions and in-country based residential sessions that provided the fellows with protected time to focus of the deliverables of the training sessions. The second part was re-scheduled to take place in 2021 with a focus on virtual clinics.
Supervisors’ Training	November 19-25	University of the Witwatersrand, South Africa	Rescheduled to be held in 2021.

Two fellowship seminars [15] that were ongoing at the onset of the pandemic – JAS1 and JAS4 - and aimed to strengthen the research capacity of newly enrolled PhD fellows and those completing their fellowships, respectively, were affected. Before the pandemic, these seminars were organised in a manner that all the fellows drawn from a specific cohort would converge in one location, in this case, at the Makerere University. The two seminars commenced before COVID was declared a global pandemic – when travel within Africa was still possible. CARTA nonetheless took standard health precautions and sensitised its community on the pandemic and related protocols, in line with WHO advisories. The sessions normally facilitated by international teaching faculty were either delivered remotely or were facilitated by African faculty members. This was not the preferred strategy since it limited fellows’ exposure to international faculty, who also provided networking opportunities – a previous strength of CARTA.

Upon declaration of the global pandemic, CARTA shortened the duration of the ongoing residential seminars, from four to two weeks, so that fellows and faculty could return to their respective countries before travel restrictions were imposed in the African region. Fellows had to complete the remaining part of their seminars using online platforms, which were either foreign to them or restrictive due to internet connectivity challenges.

The CARTA secretariat instantaneously transitioned to remote working, announced a moratorium on the disbursement of funds for new research grants and travels for fellowships, internships/short courses and conference grants, halted upcoming face-to-face governance and management meetings, training sessions and workshops and transited its activities to the virtual space. With time, and in order to optimise learning using a variety of unfamiliar delivery modes, CARTA extended the duration of the JAS from four to six weeks. In addition, CARTA invested more time and money to support the fellows and faculty to use the virtual space for learning. This created unplanned additional expenses.

CARTA JAS sessions are complex in content, being multidisciplinary and inculcating a sense of agency to affect change in the fellows. This is achieved, in part, through CARTA´s approach to pedagogy [15], in which faculty from across the CARTA partner institutions converges to facilitate in the JAS sessions. It is worth noting that planning the CARTA JAS sessions is always time intensive and at the start of the programme involved many days of joint work. Translating the JAS sessions into digital and online content to be used during the pandemic period was even more demanding, as sessional facilitators from across the partner institutions, including graduates who were ambassadors of the CARTA pedagogical approaches, were assembled virtually from across the world and in different time zones in order to refine the CARTA courses and related methodology. They were tasked with the role of developing new approaches for use in virtual learning space and identifying appropriate platforms from which to deliver the courses. This was additional work to the facilitators’ day-to-day work and their individual demands resulting from the pandemic. The entire process of revising the courses to make them current and appropriate within the virtual learning space required significant coordination that only an efficient secretariat could deliver on. It also required a committed set of faculty members, none of whom received additional salary for the extra work. While it was clear that individuals struggled to embrace the new modes of delivery within the virtual learning space, there was no option out since CARTA had to ensure that the fellows did not lose out on learning opportunities, and that they progressed adequately in their PhD journeys with minimal disruptions. Moreover, CARTA convened an emergency Vice Chancellors’ meeting early in the year, in order to assess the implication of the pandemic at programmatic and institutional levels and to solicit a common understanding on how to forge forward. A Focal Persons’ Forum was also convened early during the pandemic period in order to jointly develop a ‘new normal’ strategy, considering what was happening at the partner institutions.

#### Institutional-level experiences

The COVID pandemic-related lockdowns and travel restrictions affected each of the partner institutions distinctively. Some of the African partner universities instantaneously closed their campuses, thus limiting progress on CARTA-related institutional-based activities and projects, including progress on PhD degree programmes and related research work. Moreover, academic and research-related functions, including teaching, supervision, approvals and graduation ceremonies were restricted, and there was limited access to academic and administrative facilities, resources and services. In many cases, staff members had to initially work remotely and with limited institutional support systems, while others were actively engaged in COVID response activities as frontline workers, thus reducing their availability and support to institutional activities – all of which slowed down fellowship progression. Other institutions quickly took up virtual teaching options, kept their administration going on and conducted ethical reviews and marking of theses virtually. In many institutions’ supervisors and mentors maintained contact with their student supervisees and post-doctoral mentees, respectively. Nonetheless, CARTA consistently engaged with the partner institutions to acquaint itself with their status, experiences and challenges, with the view to work with them to progress in doctoral training and research support. Subsequently, the various universities gradually resumed their institutional-based activities and projects, but at different paces, dependent on their agility.

#### Individual/fellowship-level experiences

**Figure 3.**
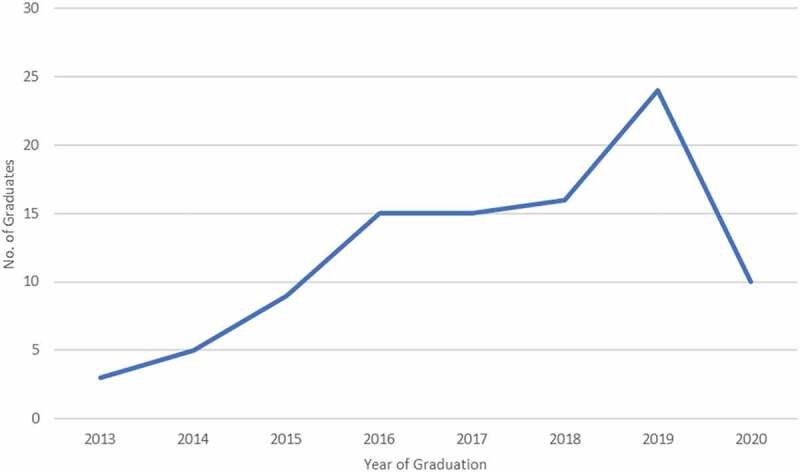
Trend in the number of CARTA PhD graduates by year (2013 to 2020).

By virtue of the philosophy of the programme, CARTA fellows are staff members of the African partner institutions. During the pandemic period, some of the fellows experienced significant disruptions to their fellowships resulting from competing interests, remote working, closure of universities, COVID-related illnesses of self or family members and engagements in institutional and national responses to the pandemic. Moreover, the fellows experienced an array of challenges ranging from balancing work and family responsibilities, including the provision of care to children and family members, insufficient internet connectivity, lack of protected time and the absence of quiet settings to meet fellowship expectations, restricted peer support on campus and among the fellows and minimal networking within the consortium. The apprehension around health, livelihood and fellowships further affected some fellows’ mental and emotional state and resulted in low productivity. All these factors may explain the reduced number of CARTA PhD graduates in 2020 (10 graduates), as compared to previous years, in which the number of graduates was gradually increasing, as shown in Figure 3.

The degree to which the pandemic impacted individual PhD fellowships depended on how far they were in their doctoral degree journey. Fellows who were in the conceptualisation and protocol development phases progressed with remote support from supervisors and programme experts. However, those who were awaiting ethical approval experienced delays due to the ripple effect of the institutional closure or disruptions. Fellows who were collecting primary data were significantly disrupted by reduced field and laboratory activities, introduction of new procedures intended to minimise the spread of COVID, extended periods of data collection and procurement of unforeseen supplies, all of which had a cost implication. Fellows who were at the final phase of their doctoral degree journey were delayed by restricted institutional-based academic and research activities. The participation of fellows in face-to-face research dissemination engagements, scientific conferences, internship programmes and short courses geared towards strengthening their professional, academic and research capacity was also inhibited, while specific post-doctoral fellowships were slowed down by external mentorship visits’ restrictions. Nonetheless, the fellows who were writing up their research results, theses and publications were able to progress in their fellowships with minimum disruptions beyond COVID-related family commitments.

### CARTA programme responses to the pandemic period

The disruptions and adjustments to the programme’s strategies were costly and initially led to delayed utilisation of planned project implementation funds and retarded progress. There was increased time and effort spent in communication, monitoring activities and brainstorming to determine the way forward during the pandemic period. As the pandemic advanced, the experiences on the ground changed and the challenges became more manageable as innovative mitigation strategies were identified and workable solutions were employed. Table 3 shows theme-based responses of CARTA to the pandemic.

**Table 3.** Thematic areas of CARTA responses to the pandemic.

Thematic Areas	CARTA Responses
Policy and resource management	Call for a moratorium on the release of funds Development of guidelines for remote working for CARTA Secretariat Development of mitigation plans, including remote working plans, weekly catch-up meetings, regular project monitoring plans and budget and activity re-adjustments, reprogramming of activities Institutional development of policies, guidelines and code of practice for online delivery of academic programs, presentation of students’ work and research supervision
Assessment, Monitoring and Evaluation	Assessment of implication of the pandemic on programmatic, fellowship and institutional activities and budgets Regular project monitoring activities Meetings to assess the situation and brainstorm on the way forward Reports on the impact of the pandemic on fellows and institutions and pandemic Status reports on the program, fellows and institutions
Communication Management	General updates to stakeholders on the status and implications of COVID-19 on the program Sensitization of the CARTA community on containment of the pandemic, including dissemination of health protocol guidelines Reassurance to stakeholders on measures being taken to stay afloat Holding meetings with CARTA stakeholders, including with vice chancellors, focal persons, fellows and funders to assess the situation and brainstorm the way forward including on institutional and fellowship activities, budgetary projections, the use of virtual platforms and re-scheduling for training and other programmatic activities Communication with fellows and institutions on program activities including cancellations, rescheduling and readjustment of activities and budgets, internet allowances Correspondences to funders on the implications of the pandemic, and soliciting guidance and consideration of extensions, bridging grants, and reprogramming and rescheduling of activities Soliciting of guidance from stakeholders on how to move forward
Supplementary investments	Procurement of Personal Protective Equipment (PPE) and supplies for fellows in research activities Enhanced access to e-learning resources Enhanced online learning infrastructural support at institutional level, including Setting up of smart classrooms to aid training sessions and online examinations Enhancement of internet support infrastructure including zoom licences, modems and internet allowance support. Strengthening of the Information and Communication Technology (ICT) capacity of staff members at partner institutions through training sessions
Execution of appropriate interventions	Adjustments to programmatic, institutional and fellowship activities, to: embrace virtual learning and academic activities, including ethical approvals, thesis defences and project supervision; consider fellowship leave of absence and project re-programming and extensions; and adapt virtual data collection techniques. Consideration of fellowship leave of absence and project extensions at fellowship, institutional and programmatic levels. Transition to virtual learning space, including the development of guidelines for virtual training, the commencement of virtual training sessions and the engagement of an e-learning expert to support the transition to virtual learning

Table 4, on the other hand, provides a summary of the monthly thematic management-related responses of CARTA to the pandemic in 2020.

**Table 4.** Summary of the monthly thematic responses in CARTA in 2020 during the pandemic period.

Thematic Response Areas	February 2020	March 2020	April 2020	May 2020	June 2020	July 2020	August 2020	September 2020	October 2020	November 2020	December 2020
Policy and Resource Management	X	X	X								
Assessment, Monitoring and Evaluation	X	X	X	X	X		X	X	X		
Communication Management	X	X		X		X	X	X	X	X	X
Supplementary investments			X	X	X	X	X	X	X	X	X
Execution of appropriate interventions		X	X	X	X	X	X	X	X	X	X

According to Table 4, policy and resource management, assessment and monitoring, and communication management responses commenced at the onset of the pandemic. Except for the policy and resource management, the other thematic responses were reported in most of the months. The policy and resource management responses were mainly reported during the first three months of the pandemic when alternative measures needed to be adopted at programmatic and institutional levels. For the most part of the year, CARTA regularly monitored the implementation of its activities and related budgets, paying attention to the implications of the pandemic on the programme. CARTA also constantly engaged with its stakeholders to identify measures to mitigate adverse effects and stay afloat amidst the pandemic, which was in line with its culture. This culture further led to ease in convening a virtual meeting with all African VCs early in the pandemic period, despite the huge demand on their time.

Supplementary investments and execution of appropriate interventions depended on assessments carried out and stakeholders’ engagements. By May 2020, CARTA had commenced discussions about the ‘new normal’, and prioritised measures that would optimise its performance, moving forward. These measures included financial support to:

- purchase internet services to fellows to engage virtually;
- pay for childcare support to allow fellows to progress in their PhD journey while their children were home;
- purchase devices or other methods to facilitate remote data collection using digital devices; and
- purchase personal protective equipment (PPE) and supplies, including masks and sanitisers, while collecting data.

Being aware of the financial implications of the mitigation measures, CARTA regularly corresponded with its funders on the implications of the pandemic on the programme and the need for extensions, mitigation grants, reprogramming and rescheduling of programmatic, institutional and fellowship activities. This was possible to achieve since the secretariat had developed an open relationship with its funders and such engagement on a range of issues was common. As a result of seamless and successful engagements, the funders permitted reprogramming of activities, re-allocation of funding budget lines and mitigation grants for:

- fellowship support, including enhanced research and stipend support, child care support and last mile thesis/dissertation support; and
- infrastructural and institutional support for the transition to the virtual space, training of institutional-based facilitators on CARTA pedagogy and related virtual learning modes, and Zoom and webinar licences.

Simultaneously, and partly as a result of engagements and commitments within the programme, partner institutions and fellows adjusted their strategies and activities. Partner institutions specifically reprogrammed their activities and:

- developed policies, guidelines and codes of practice for online delivery of academic programmes, presentation of students’ work and research supervision;
- transitioned their academic activities to virtual space;
- increased their online learning infrastructural support; and
- expanded their students’ and staff members’ access to e-learning resources and strengthened their related capacity.

On the other hand, fellows:

- reprogrammed their research activities and projects;
- adapted virtual data collection techniques;
- procured PPE and supplies to support their research projects; and
- made successful requests for project extensions and leave of absence, when appropriate.

## Discussion

We observed that as a first response to the pandemic, CARTA was immobilised, leading to the halting or rescheduling of some planned activities. This is the expected initial reaction to crises and paves the way for the assessment of situations with the aim to determine the next steps [16]. The faster an entity assesses the threatening situations, the quicker it will take to exit the immobilised state and take the necessary bold action towards either fighting back or fleeing for survival [17]. Differentiated degrees of immobilisation were encountered by partner institutions and individual fellows, all of whom responded differently and reflected different levels of agility during the pandemic period. Some institutions were prompted to adopt new ways of progressing with postgraduate training and research, including the adoption of virtual learning, and facilitation of remote research-support activities, including ethical approval and thesis/dissertation defense meetings. On the other hand, some institutions struggled to move forward due to lack of agility, reluctance and bureaucracy. Fellows’ progression was also based on individual fellows’ circumstances, the stage of their fellowships and their levels of agility. Consequently, CARTA convened regular meetings with its stakeholders in order to identify progression gaps, learn from each other and collectively work towards resolving the pandemic-instigated stalemates.

RCS initiatives need to bring on board their various stakeholders to work together towards promptly overcoming immobilisation states during crises. In the case of CARTA, there was deliberate effort made towards promptly fighting back to remain resilient, amidst the existing threats. This was witnessed through its promptness in policy and resource management, assessment and monitoring, and communication management, all of which commenced within the first month of the pandemic. As its characteristic, CARTA collaborated with its partners to assess the situation at hand and to identify solutions that would keep the programme afloat amidst the pandemic. CARTA further facilitated discussions on the status of fellowships and institutional-based strategies with the aim to establish progress and identify challenges and possible solutions. CARTA’s gendered lenses further led to the articulation of factors affecting the progress of both male and female fellows, and the prioritisation of interventions to cushion the fellows from adverse gender-related effects of the pandemic.

CARTA’s approach to the partnership has been non-hierarchical, democratic and amicable, and one that embraces both the bottom-up and top-down styles. CARTA has over the years invested in strengthening relationships with its various stakeholders who are informed of and contribute to its direction. Accordingly, the VCs of the African partner institutions promptly responded to an invitation made within the first month of the pandemic period to address the implications of COVID on postgraduate studies and research training – an act that bore fruit and propelled the fellows and institutions towards overcoming adverse effects on their progression. The response of the CARTA faculty who facilitated JAS sessions similarly indicated that the programme is jointly owned and that there is a joint responsibility to see it achieve its aims. While the secretariat had to put extra effort to ensure that response, and at times, it may have been slow, there was never a time when the secretariat was met with uttermost resistance.

In light of all the above, we summarise resilience in RCS initiatives as entailing a series of six cyclic phases comprising immobilisation, reflections, brainstorming, making resolutions, implementing interventions and recovery. These phases align with those identified by other authors who argue that the magnitude of effects of pandemic would be managed by adopting emergency management actions comprising mitigation, preparedness, responsiveness and recovery [2,5,18–24]. Moreover, we recognise the importance of adopting multiple crisis management bases to enable RCS initiatives to manoeuvre during pandemic periods. CARTA’s crisis management responses to the pandemic comprised of assessment and monitoring, communication management, policy and resource management, making investments and execution of appropriate interventions, all of which resonate with those considered under the WHO Emergency Risk Management for Health (ERMH) as bases of reference during crises [5,25].

## Conclusion

In general, the management-related strategies adopted by CARTA during the pandemic period were an extension of those employed over the years, which have contributed to its success. Investing in the administrative and institutional capacity to support RCS (in CARTA´s case a functional, competent and agile secretariat) is an essential part of strengthening research capacity and should not be considered a wasted overhead cost. Investing in the management and functioning of a consortium is also important, and time and money have to be dedicated to it.

In a recent evaluation, CARTA’s stakeholders assessed the programme as open, transparent, equitable, and one that exhibited a participatory style of leadership [9]. According to the report, CARTA has, over the past 10 years, strengthened its partnerships, promoting ownership, commitment and accountability – a factor that also contributed to its resilience during the pandemic. Moreover, CARTA has adopted a culture of mutual trust and respect for its partners, constant assessment of its progress, regular correspondence with its partners and collective decision-making and actions – all of which contributed to its resilience. CARTA’s governance structure has been profiled as robust in holding the consortium together and facilitates healthy deliberations and decision-making on the direction of the programme. Its policies and guidelines are also developed in a consultative and transparent manner, underpinned by equity, and particularly gender-lensed considerations that do not compromise on quality – all of which facilitate prompt decision-making [10]. CARTA has further invested in a vibrant, reliable and resourceful management team that effectively manages the programme and the realisation of its strategies.

As in the case of CARTA, RCS initiatives inevitably go through the critical phases of immobilisation, reflections, brainstorming, making resolutions, implementing interventions and recovery during crises. The seamless flow from one phase to another is hinged on the existence of strong partnerships, robust monitoring frameworks, effective communication management frameworks, strong governance and management strategies, effective resource management frameworks, great flexibility, long-term sustainable investments and the execution of appropriate interventions, all of which are enablers of resilience.

## Paper context

Resilient research capacity strengthening initiatives are inevitable and propel research towards authentic evidence that leads to informed decision-making, policy formation, and robust practices within the African region. This paper contributes to the knowledge based on the enablers of strong research capacity strengthening initiatives within the African context and presents management-related strategies that are key to their resilience during crises.
